# Cytokine dysregulation in children with severe neurological impairment correlates with significant clinical outcomes

**DOI:** 10.3389/fped.2025.1567221

**Published:** 2025-06-30

**Authors:** John Allen, Johana Isaza-Correa, Lynne Kelly, Ashanty Melo, Conor Power, Aoife Mahony, Denise McDonald, Eleanor J. Molloy

**Affiliations:** ^1^ Discipline of Paediatrics, Trinity College Dublin, The University of Dublin, Dublin, Ireland; ^2^ Trinity Research in Childhood Centre (TRiCC), Trinity College Dublin, Dublin, Ireland; ^3^ Trinity Translational Medicine Institute (TTMI), St James Hospital, Dublin, Ireland; ^4^ Palliative Care, Children's Health Ireland (CHI), Dublin, Ireland; ^5^ Neurodisability, Children's Health Ireland (CHI), Dublin, Ireland; ^6^ Neonatology, Children's Health Ireland (CHI), Dublin, Ireland; ^7^ Paediatrics, Coombe Hospital, Dublin, Ireland

**Keywords:** cytokine, severe neurological impairment, inflammation, lipopolysaccharide, ELISA

## Abstract

**Background:**

Children with neurological disorders have altered inflammatory responses. We aimed to describe pro-inflammatory, anti-inflammatory and hypoxia-induced cytokines in serum, at baseline, and in response to stimulation of whole blood with lipopolysaccharide, in children with Severe Neurological Impairment (SNI) compared to controls.

**Methods:**

Whole blood samples from children with SNI and healthy controls were incubated in the presence or absence of lipopolysaccharide (LPS). Serum was isolated and 12 cytokines were analysed by ELISA. Select clinical data was collected from healthcare records and correlated with cytokine results.

**Results:**

Twenty-nine children with SNI (*n* = 14) and age-matched controls (*n* = 15) were recruited. Cytokine responses to lipopolysaccharide were similar between the groups for Interferon (INF)-γ, Interleukin(IL)-18, Tumour Necrosis Factor(TNF)-β, IL-10, IL-1ra, IL-1β, IL-8, TNF-α and Vascular Endothelial Growth Factor (VEGF). Granulocyte Monocyte Colony Stimulating Factor (GM-CSF) increased in response to LPS in the control group (*p* = 0.04) but not in those with SNI (*p* = 0.07). The SNI cohort had a significantly greater increase in EPO in response to LPS than controls (*p* = 0.006). IL-6 in the SNI cohort was relatively hyporesponsive to LPS (*p* = 0.01). Correlations were found in LPS responses as follows: number of antiseizure medications and IL-1ra (*p* = 0.01) and TNF-α (*p* = 0.04); number of infections within the last year and IL-18 (*p* = 0.02); requirement for enteral feeding and IL-10 (*p* = 0.03) and EPO (*p* = 0.001); use of prophylactic antibiotics and IL-10 (*p* = 0.001); requirement for respiratory support and VEGF (*p* = 0.007).

**Conclusion:**

Children with SNI have persistent altered inflammatory responses. These alterations may contribute to tertiary neurological injury and impaired ability to respond to infection and may provide a target for immunomodulation.

## Introduction

Children with Severe Neurological Impairment (SNI) have disorders of the central nervous system (CNS) which result in significant motor and cognitive impairment as well as medical complexity (1). They are a heterogeneous group of children which includes those with a definitive (sometimes rare) diagnosis and those with none. The term overlaps with cerebral palsy (CP) and some children with CP have SNI. They are unified by the severity of their functional limitations and complex medical needs which occur as a direct result of CNS dysfunction. The CNS abnormality may be the result of many causes ranging from neuronal migration disorders to neonatal encephalopathy (NE).

Children with neurological and neurodevelopmental disorders have altered inflammatory responses (2–7). Infants with NE have alterations in serial cytokine measurements in the first week of life which correlated with mortality (5). These abnormalities appear to persist into school-age and elevated Tumour Necrosis Factor (TNF)-*β* was associated with poorer gross motor function (4). Children with one of the most well-known neurodevelopmental disorders, Down syndrome, have altered cytokine levels which may be related to their increased susceptibility to sepsis (7). Altered inflammatory responses in school-age children with cerebral palsy (CP) have also been demonstrated (2).

Cytokines are proteins secreted in response to an insult and regulate the nature and strength of the immune response by binding to cell surface receptors, initiating a signalling cascade which acts to functionally alter target cells (8). Although many cytokines may be either pro or anti-inflammatory, some have both pro and anti-inflammatory effects depending on their source, target, and timing in the immune response (9). Cytokines are, therefore, integral to the strength and quality of the immune response and a delicate balance is required to ensure health.

Abnormalities in the cytokine response to an insult may have significant impact on health-related outcomes such as morbidity related to recurrent infections or sepsis-related mortality. In CP, those with the most severe disability have been shown to have a 50% mortality by the age of 15 years (10). Death was attributed to respiratory problems in 56.8% of which 82% had pneumonia and a further 16% died from non-respiratory infections (10). Therefore, almost half of deaths in those with CP were attributable to infection. This does not consider cases where infection may have played a role in precipitating the primary cause of death e.g., in those with “>1 sufficient cause of death” recorded, half had complicated pneumonia. The reasons for this are multi-facetted, but an altered inflammatory state with altered cytokine responses may be a potential contributor.

Neurodevelopmental outcome may also be influenced by alteration of the inflammatory response. Activation of the immune system in early life is associated with several neurodevelopmental disorders including autism, schizophrenia, and CP (11). Insults such as hypoxic ischaemia can cause primary injury due to initial depletion of cellular energy stores, followed by a transient recovery and secondary reduction in high energy phosphates resulting in secondary neurological injury (12). A longer term, tertiary phase of neurological injury occurs following the initial 2 phases of cell death, during which time there is increased sensitisation to further insults (12). Inflammation, through epigenetic modifications, is postulated to play a role in tertiary neurological injury. Improving our knowledge of inflammatory responses in children with neurodevelopmental disorders could lead to discovery of potential therapeutic targets which may improve neurological outcome.

We, therefore, aimed to describe pro-inflammatory, anti-inflammatory, and hypoxia-induced cytokines in serum, at baseline, and in response to stimulation with lipopolysaccharide, in children with SNI compared to a control group of healthy children.

## Materials and methods

### Ethics and patient recruitment

Ethical approval was obtained from the Research Ethics Committee of Tallaght University Hospital (Ref: 2018/09 Chairman's Action 7). Children with SNI were identified by their primary paediatrician and had permanent “disorders of the central nervous system, resulting in motor impairment, cognitive impairment and medical complexity, where much assistance is required with activities of daily living” (13). Controls were healthy children, who were undergoing routine phlebotomy and none had an active infection, chronic disease or neurodevelopmental disorder.

The parents of children with SNI and controls were approached during a visit to the outpatient department of a paediatric hospital, provided with verbal and written information about the study and asked for consent to their child's participation. All children and young people, where appropriate, were provided with verbal and written information commensurate with their age and stage of development, following which, their assent to participation was requested. All data was collected and processed to comply with General Data Protection Regulations (GDPR).

Healthcare records were interrogated to gather a variety of clinical data, chosen based on clinical experience of issues common in children with SNI, or likely associations with altered inflammatory responses: total number of regular medications; number of antiseizure medication (ASMs); number of regular medications used to treat disorders of the gastrointestinal system (GI medications); number of infections in the past year; number of hospital admissions in the past year; requirement for prophylactic antibiotics, enteral feeding or respiratory support; and sleep score based on the Child Sleep Habit Questionnaire (CSHQ) (14).

### Blood sampling and cytokine analysis

Whole blood (3 ml) was collected in a sodium citrate anti-coagulated blood tube. Samples were incubated for 1 h at 37°C in the presence or absence of LPS(E.coli 0111:B4: SIGMA Life Science, Wicklow, Ireland) at a final concentration of 10 ng/ml. The serum was isolated and frozen at −80°C (Isotemp, Fischer Scientific) for subsequent analysis of pro and anti-inflammatory cytokines by enzyme linked immunosorbent assay (ELISA).

Twelve individual cytokines were analysed: Granulocyte Monocyte Colony Stimulating Factor (GM-CSF), tumour necrosis factor (TNF)-α, TNFβ, interleukin 1β (IL1β), interleukin 6 (IL6), interleukin 8 (IL8), interferon gamma (IFN-γ), interleukin 18 (IL18), vascular endothelial growth factor (VEGF), erythropoietin (Epo), interleukin 1 receptor antagonist (IL1ra), and interleukin 10 (IL10). A custom-made, 96 well, 10 spot, MSD® MULTI-SPOT assay plate from Mesoscale (MSD Diagnostics, USA) was utilised. The cytokines were examined via a sandwich immunoassay format where capture antibodies were coated in a patterned array on the bottom of the wells of the plate. The plates were analysed on the SECTOR Imager (Meso Scale Discovery, Rockville, MD, USA; www.meso-scale.com).

GraphPad Prism version 9.1.2 for macOS (GraphPad Software, San Diego, California, USA) and Microsoft Excel version 16.5 for Mac (Microsoft Corporation, Redmond, Washington, USA) were used for statistical analysis. Continuous data were analysed with the Shapiro–Wilk test to determine whether data were normally distributed. All normally distributed data is represented as means and standard deviations. For normally distributed data, comparison of means of 2 independent groups was performed with the student's *t*-test. The one-way ANOVA test was used to compare means of 3 or more normally-distributed independent groups. In situations where Gaussian distribution could not be assumed, data is represented as medians and 95% confidence intervals. Comparison of median values of 2 independent, non-normally distributed groups were evaluated using the Mann–Whitney *U* test. The Kruskal–Wallis test was employed to appraise the difference in median values across 3 or more groups, while Dunn's test was used for multiple comparisons. Correlation between continuous, non-normally distributed data was performed using Spearman's rank order correlation coefficient. A *p* value of <0.05 was considered significant.

## Results

Twenty-nine patients were recruited to this study. Fourteen participants (age range 1.3–15.3 years, 7 male) were recruited to the control group. There were no statistically significant differences in age or sex between the control and SNI group. Fifteen children (age range 1.8–16.5 years, 7 male) were recruited to the SNI group (Table 1).

**Table 1:** Clinical characteristics of children in the SNI group.

Variable	*n* (%)unless otherwise stated
Diagnosis	
CP	10 (66.67)
*-Dyskinetic*	*4*
*-Spastic*	*4*
*-Mixed*	*2*
Wolf Hirschhorn Syndrome	2 (13.33)
Rett syndrome	2 (13.33)
CASK mutation	1 (6.67)
Aetiology of CP	
Neonatal encephalopathy	3
Congenital brain malformation	2
Infection	2
Prematurity	2
Genetic	1
GMFCS or GMFCS equivalent (%)	
*I–III*	0 (0)
*IV–V*	15 (100)
Intellectual disability	15 (100)
Epilepsy	11 (73.3)
*Requiring 0–2 antiseizure medications*	*5*
*Requiring >2 antiseizure medications/VNS*	*6*
Visual impairment	8 (53.33)
Hearing impairment	3 (20)
Recurrent RTI requiring prophylaxis	4 (26.67)
Respiratory supportive technology	5 (33.33)
Feeding route	
Oral	6 (40)
PEG	8 (53.33)
PEJ	1 (6.67)
Number of regular medications; *mean (SD)*	6.64 (3.57)

SNI, severe neurological impairment; CP, cerebral palsy; CASK, calcium/calmodulin dependent serine protein kinase; GMFCS, gross motor function classification system; VNS, vagal nerve stimulator; RTI, respiratory tract infection; PEG, percutaneous endoscopic gastrostomy; PEJ, percutaneous endoscopic jejunostomy.

**Figure 1:**
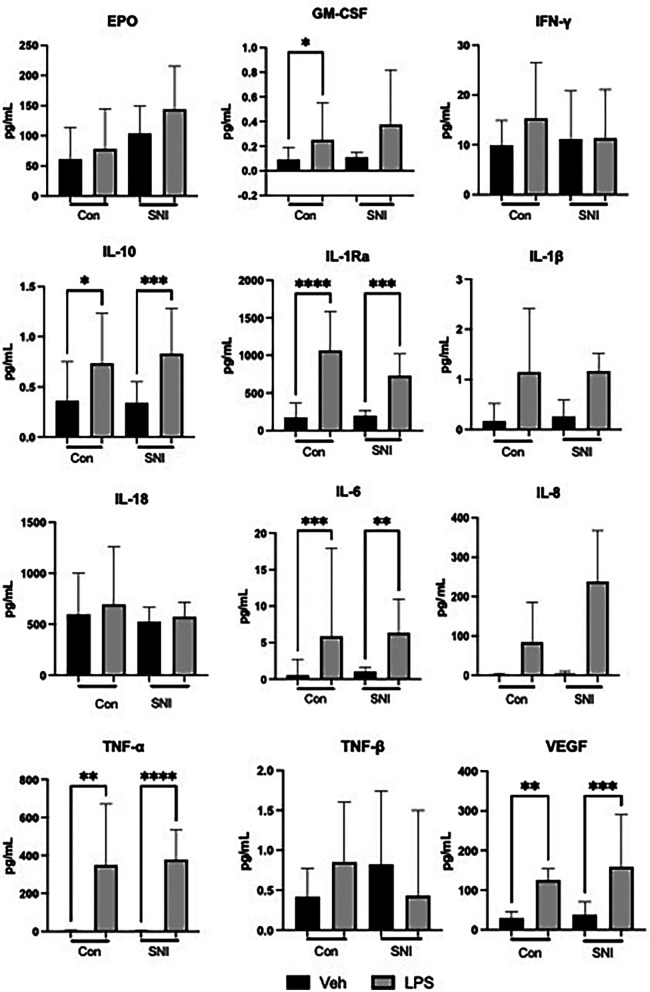
Cytokine values (pg/ml) at baseline and following stimulation for 1 h with lipopolysaccharide (LPS; 10 ng/ml). Data are displayed as Median [95%CI] and were analysed using the Kruskal–Wallis test. *p ≤ 0.05; **p ≤ 0.01; ***p ≤ 0.001; ****p ≤ 0.0001; Veh, vehicle.

There were no significant differences in baseline values between the control and SNI cohorts (Table 2; Figure 1). No significant increase in cytokine level was seen in either the control or SNI cohorts for EPO (*p* > 0.99; *p* = 0.71), INF-γ (*p* = 0.84; *p* > 0.99), IL-18 (*p* > 0.99; *p* > 0.99) or TNF-β (*p* = 0.58; *p* > 0.99; Table 2; Figure 1).

**Table 2:** Cytokine values (pg/ml) at baseline and following stimulation with 1 ul lipopolysaccharide (LPS; 10 ng/ml) for every 100 ul of peripheral blood for 1 h.

Cytokine	Control (*n* = 14)		SNI (*n* = 15)					
	Veh	LPS	Veh	LPS	*p* ^a^	*p* ^b^	*p* ^c^	*p* ^d^
GM-CSF	0.09 [0.05, 0.19]	0.25 [0.16, 0.55]	0.11 [0.08, 0.15]	0.375 [0.12, 0.82]	>0.99	**0**.04*	0.07	>0.99
IFN-γ	9.925 [6.62, 14.89]	15.33 [8.8, 26.48]	11.17 [6.8, 20.81]	11.34 [8.07, 21.09]	>0.99	0.84	>0.99	>0.99
IL-10	0.36 [0.25, 0.75]	0.735 [0.49, 1.23]	0.34 [0.29, 0.55]	0.83 [0.63, 1.28]	>0.99	**0**.02*	**0**.0005***	>0.99
IL-18	597 [464.2, 1,003]	696.1 [498, 1,260]	526.6 [343.3, 666.5]	574 [500.4, 716.4]	0.37	>0.99	>0.99	>0.99
IL-1ra	175.9 [125.9, 364.3]	1,063 [398.4, 1,585]	196.9 [162, 268.7]	730.6 [571, 1,020]	>0.99	**<0**.0001****	**0**.0007***	>0.99
IL-1β	0.17 [0.05, 0.52]	1.145 [0.48, 2.41]	0.26 [0.12, 0.59]	1.17 [0.7, 1.52]	>0.99	**0**.0008***	**0**.0023**	>0.99
IL-6	0.54 [0.33, 2.74]	5.865 [2.76, 17.96]	1.02 [0.62, 1.62]	6.34 [3.74, 10.97]	>0.99	**0**.0002***	**0**.002**	>0.99
IL-8	3.71 [2.54, 5.49]	84.32 [34.2, 186]	5.16 3.41, 10.53]	238.6 [172.9, 367.8]	>0.99	**0**.001***	**<0**.0001****	0.51
TNF-α	3.265 [2.2, 9.19]	349.3 [132.2, 670.2]	2.83 2.4, 4.06]	378.2 [288.2, 537.6]	>0.99	**0**.001***	**<0**.0001****	>0.99
TNF-β	0.42 [0.21, 0.77]	0.85 [0.34, 1.6]	0.825 [0.3, 1.74]	0.43 [0.11, 1.5]	>0.99	0.58	>0.99	>0.99
EPO	61.39 [47.54,113.9]	78.2 [53.01,145]	103.8 [71.34, 149.5]	144.1 [96.2, 215.3]	0.60	>0.99	0.71	0.09
VEGF	29.55 [23.11, 46.06]	125.7 [94.48, 153.5]	37.9 [29.28, 70.96]	158.4 134.9, 290.2]	>0.99	**0**.001***	**0**.0004***	0.91

Significant *p* values are highlighted in bold.

* Represents a *p* value ≤0.05; **Represents a *p* value ≤0.01; ***Represents a *p* value ≤0.001; ****Represents a *p* value ≤0.0001; SNI, severe neurological impairment; Veh, vehicle; ^a^Baseline cytokine levels between controls and SNI.

^b^ Control cytokine levels before and after stimulation with LPS.

^c^ SNI cytokine levels before and after stimulation with LPS.

^d^ Cytokine levels in control and SNI groups following LPS stimulation; EPO, erythropoeitin; GM-CSF, granulocyte-monocyte colony stimulating factor; INF-γ, interferon gamma; IL, interleukin; TNF, tumour necrosis factor; VEGF, vascular endothelial growth factor.

Significant increases were seen after addition of LPS in both the control and SNI groups in the following cytokines, indicating an appropriate response to LPS stimulation: IL-10, IL-1ra, IL-1β, IL-6, IL-8, TNF-α and VEGF (Table 2; Figure 1). The level of GM-CSF increased significantly in the control group (*p* = 0.04) but not in the group of children with SNI (*p* = 0.07).

**Figure 2:**
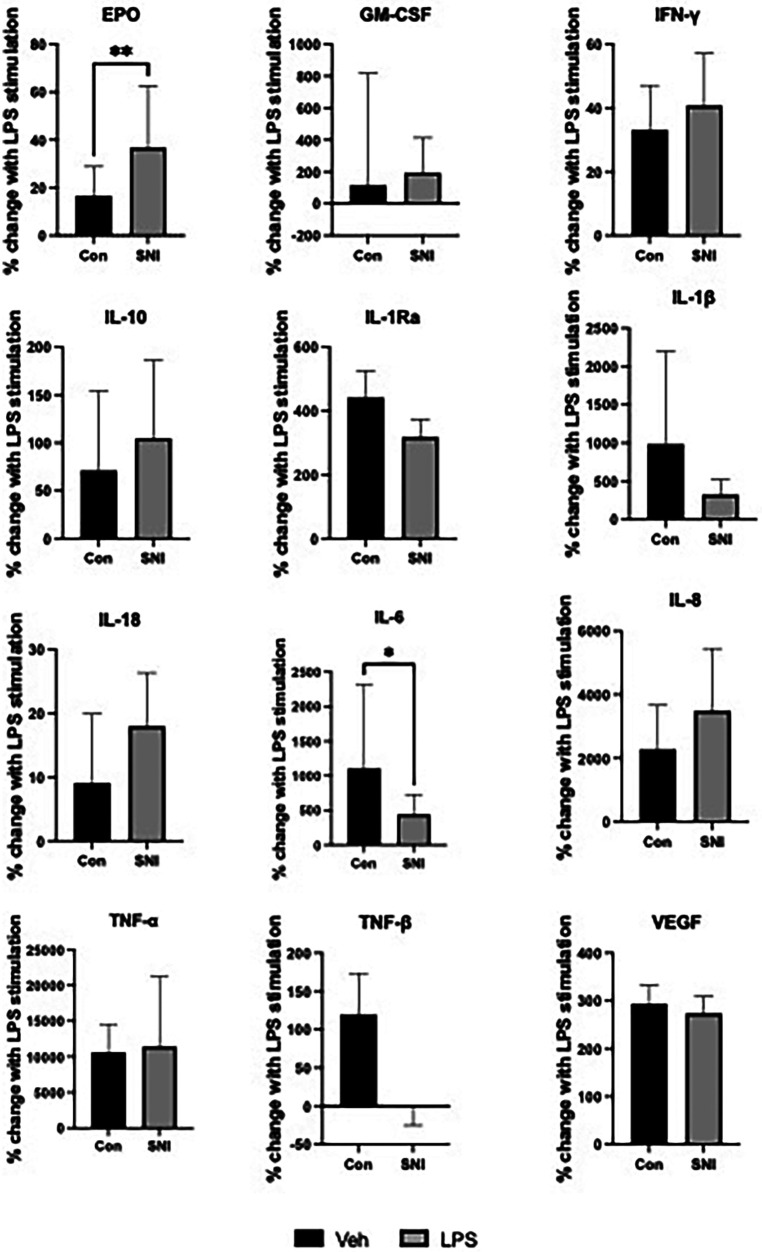
Percentage change in cytokine values following stimulation for 1 h with lipopolysaccharide (LPS; 10 ng/ml). Data are displayed as Median [95%CI] except INF-γ, IL1-Ra, TNF-β and VEGF which are displayed as Mean (SD). Normally distributed data were analysed using the student t test while the Mann–Whitney *U* test was used to analyse data where normal distribution could not be assumed. *p ≤ 0.05; **p ≤ 0.01; Veh, vehicle; EPO, erythropoietin; GM-CSF, granulocyte-monocyte colony stimulating factor; INF-γ, interferon gamma; IL, interleukin; TNF, tumour necrosis factor; VEGF, vascular endothelial growth factor.

The SNI cohort had a relatively larger increase in EPO in response to LPS than the comparison group with a median increase of 36.77% (95% CI 22.16–62.42) vs. 16.53% (95% CI 7.44–28.86) respectively (*p* = 0.0068). IL-6 in the SNI cohort was relatively hyporesponsive to LPS with a median increase of 443% (95% CI 216.9–728.7) vs. 1106% (95% CI 541.9–2,320) respectively (*p* = 0.012). There were no significant differences in the relative change of cytokine level before or after LPS stimulation for any of the other markers studied (Table 3; Figure 2).

**Table 3:** Percentage change in cytokine levels following stimulation for 1 h with lipopolysaccharide (LPS; 10 ng/ml).

Cytokine	Control (*n* = 14)	SNI (*n* = 15)	*p* value
EPO	16.53 [7.44, 28.86]	36.77 [22.16, 62.42]	0.0068**
GM-CSF	115.4 [−4.76, 816.7]	194.8 [9.09, 412.5]	0.9286
INF-γ mean(SD)	33.1 (±48.52)	40.77 (±61.01)	0.7293
IL-10	71.19 [25.64, 154.1]	104.9 [63.1, 186.2]	0.1225
IL-18	9.095 [3.03, 19.88]	18 [7.46, 26.36]	0.1456
IL-1ra mean(SD)	442.9 (±307.4)	318.7 (±212.7)	0.2142
IL-1β	986.7 [121.2, 2,196]	325 [147.3, 522.2]	0.054
IL-6	1,106 [541.9, 2,320]	443.1 [216.9, 728.7]	0.012*
IL-8	2,288 [1,246, 3,664]	3,495 [2,133, 5,448]	0.23
TNF-α	10,645 [2,562, 14,565]	11,417 [8,159, 21,269]	0.50
TNF-β mean(SD)	119.9 (±177.5)	−1.46 (±78.29)	0.051
VEGF mean(SD)	293.7 (±144.7)	273.8 (±135.8)	0.70

Significant *p* values are highlighted in bold.

Data are displayed as Median [95%CI] except INF-γ, IL1-Ra, TNF-β and VEGF which are displayed as Mean (SD). *p ≤ 0.05; **p ≤ 0.01; Veh, vehicle; EPO, erythropoietin; GM-CSF, granulocyte-monocyte colony stimulating factor; INF-γ, interferon gamma; IL, interleukin; TNF, tumour necrosis factor; VEGF, vascular endothelial growth factor.

### Correlation of cytokines with other markers of health

Total number of medications, number of GI medications and number of hospital admissions in the past year did not correlate with LPS responsiveness of any of the cytokines which were analysed. Number of ASMs correlated positively with responsiveness of IL-1ra (*r* = 0.6217, 95% CI 0.1443–0.8644, *p* = 0.0155) and negatively with TNF-α (r = 0.5212, 95% CI −0.0046 to −0.8212, *p* = 0.0487). A significant negative correlation was found between number of infections within the last year and response of IL-18 to LPS (*r* = −0.5844, 95% CI −0.8487 to −0.0864, *p* = 0.0253). Requirement for enteral feeding was associated with a lower response of IL-10 (OR 0.87, 95%CI 0.67–0.99, *p* = 0.0342) and EPO (OR 0.8586, 95%CI 0.68–0.96, *p* = 0.0012) to LPS. Use of prophylactic antibiotics was associated with lower IL-10 (OR 0.83, 95% CI 0.63 to 0.95, *p* = 0.0011) LPS responsiveness. Requirement for respiratory support was associated with lower VEGF (OR 0.98, 95% CI 0.959–0.996, *p* = 0.0079). Sleep score, as measured on the Children's Sleep Habit Questionnaire (CSHQ), was negatively correlated with percentage change in EPO following stimulation with LPS (*r* = −0.6331, 95%CI −0.8976 to −0.033, *p* = 0.0413).

## Discussion

We have shown alterations in pro and anti-inflammatory cytokines in children with SNI. This suggests an underlying abnormal inflammatory state which may have important effects on morbidity and mortality in this population. In particular, this may result in significant impairment of immune function resulting in increased susceptibility to infection. There may also be a role for altered inflammatory responses in leading to tertiary neurological injury.

None of the cytokines measured were significantly different at baseline compared to the control group. This is similar to the findings by Zareen et al. in the CP population (2), although they reported a higher EPO at baseline in the children with CP. However, our cohort of children did demonstrate a significantly greater EPO response to LPS stimulation than the control population. EPO is an acidic glycoprotein essential to the production of red blood cells. The kidneys are the main source of production in response to reduction in the partial pressure of oxygen (pO2). Subsequently, however, EPO expression has been noted in several other organs including the liver, brain, spleen, and lung (15). Non-erythropoietic effects of EPO have been suggested in immune modulation, regulation of vascular tone in response to an acute ischaemic event, muscle development and remodelling, metabolic homeostasis, and bone formation and homeostasis (16). Its role in immune modulation and its anti-apoptotic effects, in particular, have led to much research into potential roles in neuroprotection and treatment of sepsis (17–20). A meta-analysis of EPO in NE suggested a reduction in risk of brain injury, CP, and cognitive impairment (21). However, the HEAL randomised controlled trial of high dose EPO for perinatal asphyxia and encephalopathy in term infants did not lower risk of death or neurodevelopmental impairment and was associated with a higher risk of adverse events (22). Similarly, in preterm infants, a large randomised double-blind trial (the PENUT trial) found that EPO did not reduce the occurrence of severe neurodevelopmental impairment or death at 2 years (23). Elevated EPO concentrations are associated with poorer neurodevelopmental outcome and increased mortality in preterm infants and those with NE (24, 25). The clinical significance of increased LPS-responsiveness of EPO in our patients is difficult to ascertain and will require further prospective study including detailed sleep studies.

In both the HEAL and PENUT study the early outcomes at 2 years may be insufficient to see longer term neuroprotective effects (26). At later timepoints from 3 to 10 years improved neurological and cognitive outcomes have been reported with EPO (27–29).

We hypothesise that many children with SNI have obstructive sleep apnoea and this may contribute to the increased EPO and ability to upregulate following LPS. We have seen similar LPS-induced increases in EPO in infants with neonatal encephalopathy in the first days of life consistently (24). The degree of elevation of EPO was associated with the severity of NE and degree of hypoxia exposure (30) Sleep disruption is common in children and adults with SNI and (31)/or cerebral palsy. We have also demonstrated dysfunction sleep in childhood post NE (32, 33). However, a direct comparison between sleep studies and serum erythropoietin and LPS responses is required and may validate EPO as a biomarker to indicate the need for a sleep study in children with SNI. We have also demonstrated hyporesponsiveness of IL-6 to stimulation with LPS. In both controls and children with SNI, there was a significant rise of IL-6 following endotoxin exposure compared to baseline levels. However, in the SNI population the relative rise was less than that of controls. This is in keeping with the findings of Zareen et al. that children with CP are relatively IL-6 hyporesponsive (2). A systematic review of inflammatory biomarkers in children with CP found that higher levels of IL-6 are associated with abnormal neurological outcome (34) and IL-6 polymorphisms have been shown to increase the risk of developing CP (35, 36). Altered inflammation in early life has a programming effect and can alter inflammatory responses later in life (37). IL-6 is a significant component of the systemic inflammatory response to infection (38) and correlates with survival of patients with sepsis (39). Critically ill adult patients admitted to ICU have been found to have lower production of IL-6 in response to LPS. This, amongst other immune markers, may indicate an impaired inflammatory response in the early phase of critical illness (60). Cetiner et al. reported a decreased IL-6 level, reduced TNF-α, and increased IL-10 and IL-4 in children with Down syndrome. This anti-inflammatory picture may explain the higher rate of respiratory tract infections in this cohort (61). IL-6 hyporesponsiveness, as seen in our study, may affect the ability of children with SNI to respond adequately to infection. Future cohorts will prospectively measure IL-6 in these children and following outcomes such as number of infections per year, requirement for antibiotic treatment, infection-related hospitalisations, intensive care admissions related to infectious causes, sepsis, and mortality.

We correlated cytokine responses in the SNI group with several other health-related outcome measures. The CSHQ score and requirement for respiratory support were positively correlated with EPO and VEGF respectively. Both cytokines are sensitive to hypoxia and have been shown previously to be raised in obstructive sleep apnoea (40, 41).

We have demonstrated that recurrent respiratory tract infections were negatively correlated with IL-18 responses. IL-18 is a pro-inflammatory cytokine which induces INF-γ, nitric oxide and reactive oxygen species in phagocytes and thus plays a role in clearance of various micro-organisms. It also activates CD8+ *T* cells which are involved with viral clearance. On the other hand, IL-18 hyper-responsiveness has been implicated as a contributory factor to the cytokine storm and increased mortality in conditions such as sepsis and Coronavirus Disease 2019 (COVID-19) (42). Further research is required to determine the role of IL-18 in respiratory tract infections in children with SNI and whether it may prove useful as a therapeutic target.

Use of prophylactic antibiotics was correlated with IL-10 levels. Azithromycin was the most commonly used prophylactic antibiotic in our population of children with SNI. Macrolide antibiotics are known to have bacteriostatic activity but also exert an effect through their immunomodulatory properties (43). IL-10 is a predominantly anti-inflammatory cytokine, and it is probable that the anti-inflammatory properties of azithromycin are, in part, exerted through modulation of IL-10 production (44).

Requirement for enteral feeding, such as with percutaneous endoscopic gastrostomy (PEG) or percutaneous endoscopic jejunostomy (PEJ), was correlated with lower IL-10 and EPO responsiveness. Research is lacking in this area but, in contrast to our findings, a randomised double-blind trial in adults with acute pancreatitis showed increased IL-10 levels in those administered enteral nutrition vs. those who received parenteral nutrition (45). In neonates, delayed enteral feeding is correlated with decreased IL-10:IL-8 ratios (46).

Increasing number of ASMs was correlated with higher IL-1ra and with lower TNF-α. Epilepsy and seizures are known to be associated with a pro-inflammatory state (47–49). The anti-inflammatory effect of ASMs on cytokines has been well demonstrated and has been proposed as one of the mechanisms of action of these drugs (47, 50). IL-1ra has powerful anti-convulsant effects (51) and potential as a therapeutic target in neuroprotection and seizure prevention (52). Similar to our findings, TNF-α was found to be decreased in response to 9 different ASMs in *in-vitro* studies (50). IL-1ra is an anti-inflammatory cytokine, while TNF-α is considered predominantly pro-inflammatory. Thus, the overall association appears to anti-inflammatory with increasing numbers of ASMs. This may be considered a beneficial side-effect of antiseizure medications and indeed, in several animal studies ASMs have been shown to have beneficial effects in systemic inflammatory states such as sepsis (53–58).

The main limitation of this study is the small sample size. However, our findings are in keeping with previous studies in the area and serve to confirm these results. There is a significant paucity of research regarding cytokine and immune dysregulation in children with neurodevelopmental disorders, particularly those at school-age. A better understanding of cytokine dysregulation has the potential to lead to an enhanced array of therapeutic options which may ultimately lead to improved health-related outcomes.

There is growing evidence that children with CP and SNI have persistent altered inflammatory responses. These alterations may lead to tertiary neurological injury and impaired ability to respond to infection, warranting further large longitudinal studies. Further understanding of immune function in children with SNI has significant potential to improve outcomes through immunomodulation.

## Data Availability

The raw data supporting the conclusions of this article will be made available by the authors, without undue reservation.
